# Emergency department crowding and length of stay before and after an increased catchment area

**DOI:** 10.1186/s12913-019-4342-4

**Published:** 2019-07-22

**Authors:** Ida Mentzoni, Stig Tore Bogstrand, Kashif Waqar Faiz

**Affiliations:** 1 Emergency Department, Akershus University Hospital, Lørenskog, Norway; 2 Lovisenberg Diaconal University College, Oslo, Norway; 3 Department of Forensic Sciences, Section of Drug Abuse Research, Oslo University Hospital, Oslo, Norway; 4 Institute of Health and Society, University of Oslo, Oslo, Norway; 5 Health Services Research Unit, Akershus University Hospital, PO Box 1000, N-1478 Lørenskog, Norway; 6 Department of Neurology, Akershus University Hospital, Lørenskog, Norway

**Keywords:** Emergency department, Hospital, Length of stay, Crowding, Triage

## Abstract

**Background:**

Emergency department (ED) crowding and prolonged length of stay (LOS) are associated with delays in treatment, adverse outcomes and decreased patient satisfaction. Hospital restructuring and mergers are often associated with increased ED crowding. The aim of this study was to explore ED crowding and LOS in Norway’s largest ED before and after an increased catchment area.

**Methods:**

The catchment area of Akershus University Hospital increased by approximately 150,000 inhabitants in 2011, from 340,000 to 490,000. In this retrospective study, admissions to the ED during a six-year period, from Jan 1st 2010 to Dec 31st 2015 were included and analyzed.

**Results:**

A total of 179,989 admissions were included (51.0% men). The highest occupancy rate was in the age group 70–79 years. Following the increase in the catchment area, the annual ED admissions increased by 8343 (40.9%) from 2010 to 2011, and peaked in 2013 (34,002). Mean LOS increased from 3:59 h in 2010 to 4:17 in 2012 (highest), and decreased to 3:45 h in 2015 after staff, capacity and organizational measures. In 2010, 37.9% of the ED patients experienced crowding, and this proportion increased to between 52.9–77.6% in 2011–2015. Crowding peaked between 4 and 5 PM.

**Conclusions:**

LOS increased and crowding was more frequent after a major increase in the hospital’s catchment area in Norway’s largest emergency department. Even after 5 years, the LOS was higher than before the expansion, mainly because of the throughput and output components, which were not properly adapted to the changes in input.

**Electronic supplementary material:**

The online version of this article (10.1186/s12913-019-4342-4) contains supplementary material, which is available to authorized users.

## Background

Emergency department (ED) crowding is an issue of great concern worldwide.

Crowding is often defined as a situation in which the identified need for emergency services exceeds available resources for patient care in the ED, hospital, or both [1], and leads to adverse outcomes and reduced quality [2].

Length of stay (LOS) is not a direct measure of crowding, but it is an important indicator [3] and a tool to monitor emergency care quality [4]. Increased LOS has been associated with delays in treatment, adverse outcomes and decreased patient satisfaction [5–8].

The ED serves as a buffer for overstretched hospital wards, but crowding stretches the resources of the ED, which in turn can make it difficult for the ED to deliver the necessary and safe care to its patients [2, 9]. Although there seems to be a common understanding regarding the concept of crowding, and several studies have been performed on the subject, there is no standard measure of ED crowding [10].

Asplin et al. [9] have presented one commonly accepted conceptual framework for studying ED crowding: the input – throughput – output model. *The input component* in the model includes any event or system feature, which contributes to the demand for the ED services. It consists most importantly of emergency care, but also unscheduled urgent care (because of insufficient capacity in other parts of the acute care system) and safety-net care. In *the throughput component*, the internal ED processes are assessed and modified in order to improve efficiency and effectiveness. In this component, patient LOS is seen as a contributing factor to ED crowding, and is divided into a *first phase* (triage, room placement and the initial evaluation) and a second phase (diagnostic work up and treatment). Effective ED triage and room placement within some minutes after patient arrival, and an initial physician evaluation thereafter, reduces the LOS. Nevertheless, diagnostic work up and treatment constitutes the majority of the LOS. Both phases in the throughput component are influenced by nurse and physician staffing, but also by efficient use of diagnostic testing, and the quality of the documentation and communication systems in the ED. The final part of the model, *the output component*, concerns the accumulation of admitted and discharged patients, mostly because of the inability to move patients from the ED to a hospital ward, as inpatient beds are not available. This is often referred to as boarding of inpatients in the ED.

The Norwegian health care system differs somewhat from other health care services. About all Norwegian hospitals are public and are financed by taxation and co-payment. All Norwegian healthcare takers have their own general practitioner (GP) and are covered by The National Insurance Scheme (Folketrygden). All municipalities are obliged to provide out-of-hours emergency services, either located in the municipality or organized in cooperation with other municipalities. The out-of-hours emergency services are staffed with emergency physicians (EPs). All patients admitted to the hospital ED need a referral from a physician, usually their GP or an EP, or are transported directly by the emergency medical services (EMS) in acute conditions. In case of medical emergency, the patients can call the emergency medical dispatch (113), which is staffed with nurses and paramedics with special training. The ambulances are staffed with paramedics and medical emergency technicians [11]. This comprehensive pre-hospital organization results in a high level of selection of patients who present to the ED. In Norway, the specialty *Emergency Physician* is not yet established, and patients who come to a hospital ED, do not meet a “generalist” but a resident. The type of resident/ specialty is dependent on the pre-hospital admission diagnosis/ chief complaint. Physicians, who evaluate the patients in the ED, are organized in the main specialties Internal Medicine, Surgery, Neurology and Orthopedics with separate on-call systems.

Hospital restructuring and mergers are associated with increased ED crowding, even after controlling for utilization and patient demographics [12]. Restructuring resulting in reductions in hospital resources is often not proceeded slowly enough to allow time for monitoring and evaluation [12]. Crowding is related to the capacity of the ED, and local thresholds have to be used in order to define crowding. Information of arrival time and LOS in the emergency department is often available through the hospital information system and it is therefore possible to study changes in patient’ admissions to the ED over time.

Akershus University Hospital, which is Norway’s largest emergency care hospital, is located about 20 km outside of the capitol Oslo, and the hospital’s catchment area consists of a mixed urban and rural population. The Ministry of Health and Care Services has the supervisory responsibility for all hospitals in Norway, and the state owns the public hospitals, which are organized into four regional health authorities (RHAs). In 2009, the South-Eastern RHA decided to merge four hospitals in Oslo, namely Ullevål, Rikshospitalet, Radiumhospitalet and Aker. The four hospitals were merged to form Oslo University Hospital. The hospitals had overlapping functions, and the arguments in favor of merging were economic (reduced costs) and increased quality. In 2010, the South-Eastern RHA decided on closure of the former Aker University Hospital, and the about 150,000 inhabitants which had Aker as their local hospital, were transferred to Akershus University Hospital. Thus, the catchment area of Akershus University Hospital increased by 44% from Jan 1st 2011, from 340,000 to 490,000, the latter approximately 10% of the Norwegian population. The hospital was already, before the increased catchment area, struggling with bed capacity and had a high occupancy level.

The aim of this study was to explore ED crowding and LOS in Norway’s largest ED before and after an increased catchment area.

## Methods

This retrospective study was conducted at Akershus University Hospital. Data were collected from the hospital’s electronic clinical database. The consecutive sample consists of all admissions to the ED during a six-year period, from Jan 1st 2010 to Dec 31st 2015.

Regarding the input – throughput – output model [9], the increased catchment area resulted in increased patient volume, and the input component could be influenced by e.g. increased travel distances to the ED for many residents (the individual’s likelihood of seeking care). The throughput and output components were noticeably affected by the increased catchment area, and changes are summarized in Table 1. Regarding the first phase of the throughput component, triage using the Manchester Triage System (MTS) was introduced only in 2013, and a dedicated area with beds for triage and initial evaluation was used from 2014. Regarding the second phase, some extra examination rooms were added in order to increase the ED physical capacity, and nurse and physician staffing was gradually increased. Concerning the output component, inpatient beds were increased.

**Table 1:** Akershus University Hospital Emergency Department capacity, staffing, and crowding definitions

	2010	2011	2012	2013	2014	2015	∆ 2010–2011 (%)	∆ 2010–2015 (%)
Examination rooms	19	21	21	21	24	24	2 (10.5)	5 (26.3)
Examination beds	25	34	34	34	43	43	9 (36.0)	18 (72.0)
Physician shifts per day	21.0	24.0	25.6	29.9	37.5	33.1	3 (14.3)	12.1 (57.6)
Nurse shifts per day	37.5	46.3	48.6	47.6	52.2	46.2	8.8 (23.5)	8.7 (23.2)
Somatic inpatient beds^a^	496	616	616	601	601	598	120 (24.2)	102 (20.6)
Average occupancy rate for hospital somatic inpatient beds (%)^a^	91.0	98.1	93.3	95.7	95.5	93.0	7.1	2.0
Crowding definitions:								
- Normal operational level	< 25	< 35	< 35	< 35	< 35	< 35		
- High operational level	25–34	35–44	35–44	35–44	35–44	35–44		
- Critical operational level	≥ 35	≥ 45	≥ 45	≥ 45	≥ 45	≥ 45		

^a^Total inpatient beds excluding psychiatry

Patient demographics included gender and age. Age was analyzed in 10-year groups. Arrival date, day and time, and discharge date, day and time from the ED were recorded.

Patients under the age of 18 and patients admitted because of psychiatric conditions are not admitted through the ED and could thus not be identified in the electronic clinical database. Thus, children and adolescents under 18 years were excluded from the study.

A total of 189,706 admissions were registered in the electronic clinical database. For every patient, type of specialty needed for evaluation was categorized as i) Internal medicine; ii) Surgery; iii) Orthopaedics; and iv) Neurology. If specialty was changed during the ED stay, the specialty on ED discharge was recorded.

Patients seeking help for gynaecological, obstetric and ear, nose and throat (ENT) were excluded from the study (*n* = 9717) as they primarily are not evaluated in the ED, but at the outpatient clinics.

### Length of stay

LOS was calculated as the difference between the inflow time and the outflow time.

### Crowding

As there is currently no unified definition of ED crowding, the hospital’s internal definition of operational levels was used to define crowding (Table 1). Crowding was defined by two thresholds: i) *high operational level* if the total number of patients present in the ED exceeded 25 (2010)/ 35 (from 2011), and ii) *critical operational level* if the total number of patients present in the ED exceeded 35 (2010)/ 45 (from 2011). A *normal operational level* was thus defined as < 25 (2010)/ 35 (from 2011) patients in the ED. According to a hospital procedure, different actions and measures were activated when the two thresholds were reached. Examples of measures were: a status meeting between involved health care personnel, to retrieve additional nurses and physicians (including senior consultants) from hospital wards to the ED, use the 23-beds Clinical Decision Unit located next to the ED as an extended emergency department, and that patients who were waiting for an inpatient bed were transported to the ward within 15 min regardless of the ward capacity.

Crowding (high or critical operational level) was measured by detecting the number of patients who were present in the ED at every hour (e.g. 12 o’clock, 13 o’clock etc.) in the time period between 12.00–22.00 (11 measurements). A percentage was then calculated based on the number of patients who experienced crowding for each time measurement.

### Statistics

Categorical variables are presented as absolute values and percentages. Continuous variables are presented as mean and standard deviation (SD) and median and quartiles.

In order to identify factors associated to ED LOS, a multiple linear regression model with LOS as the dependent variable, and admission year, admission day, age group, gender and specialty as independent variables, was estimated. Due to a non-linear relationship to LOS, all independent variables were entered into the model as dummy variables with the year 2010, Monday as admission day, the youngest age group (< 20 years), male gender and internal medicine as reference categories. The results are presented as regression coefficients with 95% confidence intervals (CI) and *p*-values. The regression coefficients are to be interpreted as average difference in LOS between reference and any other category of a particular variable.

The statistical analyses were performed in SPSS version 25. All tests were two-sided and the results with *p*-values below 0.05 were considered statistically significant.

### Ethics

According to Norwegian law on medical research, quality improvement and assurance projects do not require an approval by the Regional Ethics Committee, nor a written patient consent. The study was approved by the local Data Protection Authorities (ref. no. 15–148). Data were anonymized and secured on a research server at Akershus University Hospital.

## Results

A total of 179,989 admissions were included in the study (51.0% men). Because of the increase in the catchment area from 2010 to 2011, ED examination rooms and beds increased by 10.5 and 26.0%, respectively (Table 1). Nurse and physician staffing increased by 23.5 and 14.3%, respectively, while the number of inpatient beds increased from 496 to 616 (24.2%) (Table 1). Nurse and physician staffing increased gradually over the next years, but was then reduced in 2015.

**Fig. 1:**
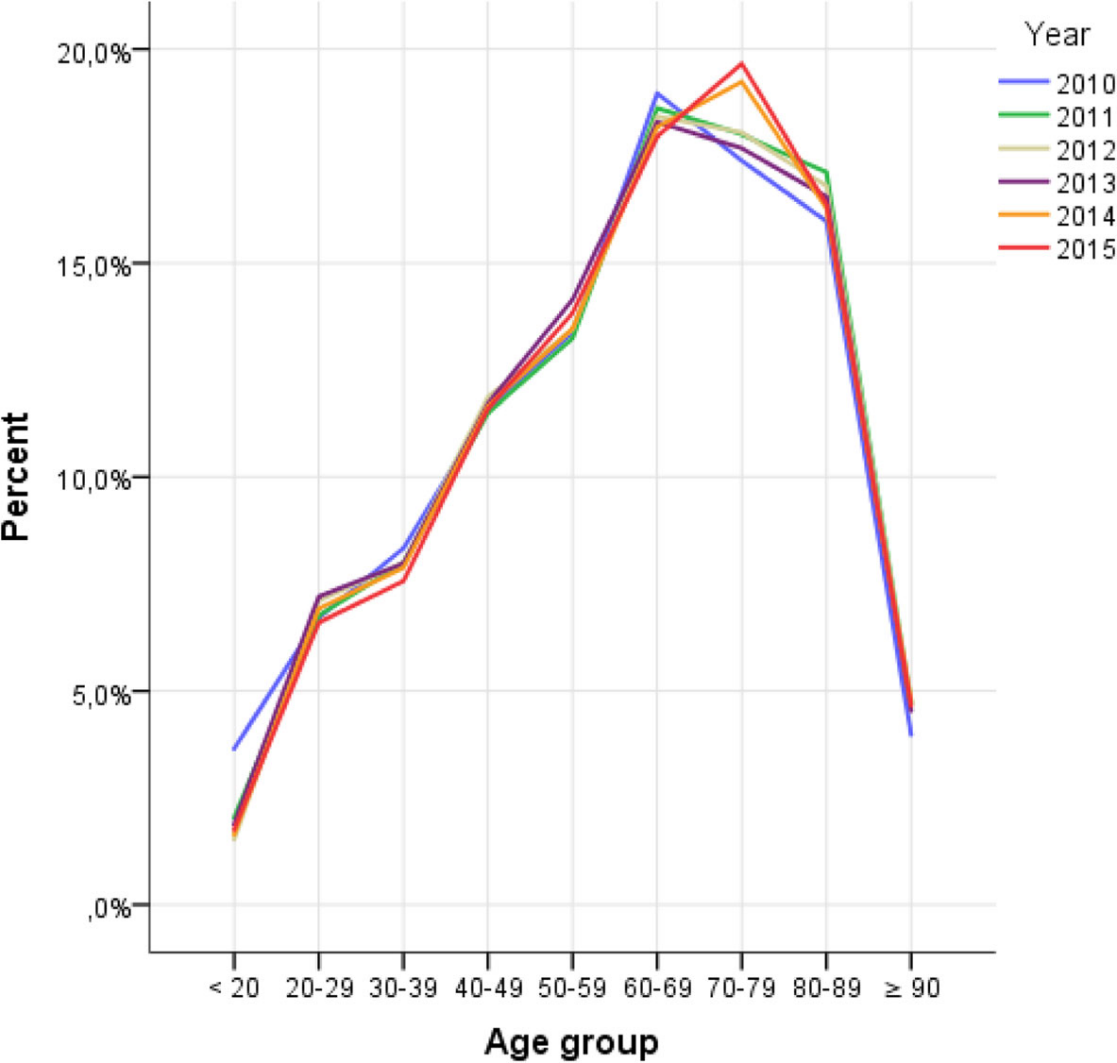
Emergency department patients ≥ 18 years, age distribution (10-years groups) per year, 2010–2015, *n* = 179,989

**Fig. 2:**
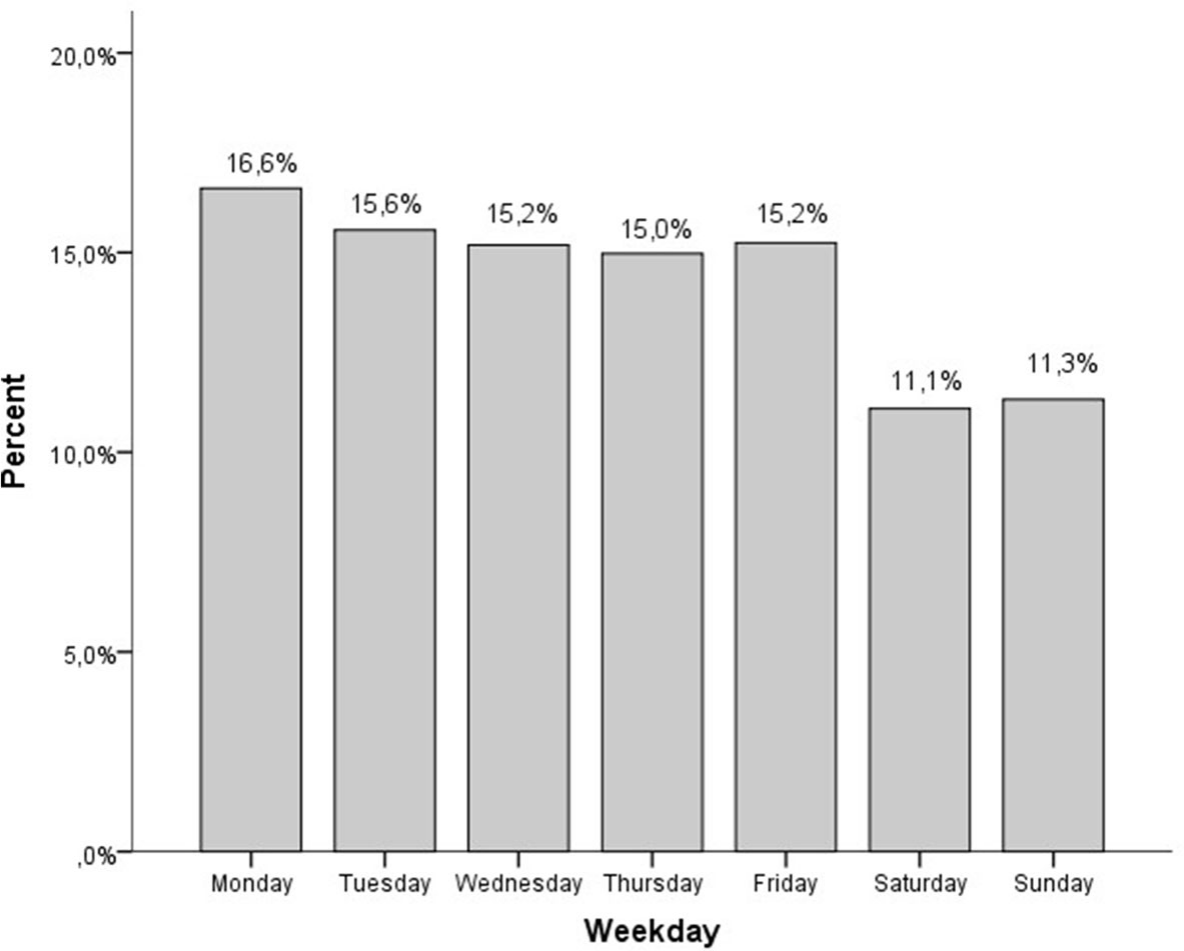
Emergency department patients ≥ 18 years, weekday distribution, merged for the years 2010–2015, *n* = 179,989

The annual ED admissions increased by 8,343 (40.9%) from 2010 to 2011. All specialties experienced a varied degree of increase in admissions, between 29.1% (orthopaedics) and 46.5% (neurology). The number of admissions peaked in 2013 (34,002). The number of admissions are shown in Table 2. The highest occupancy rate was in the age group 70–79 years (Fig. 1). The highest occupancy rate was on Mondays (16.6%) (Fig. 2).

**Table 2:** Admission data 2010–2015

	Total	2010	2011	2012	2013	2014	2015	∆ 2010–2011	∆ 2010–2015
Patients	179,989	20,395	28,738	32,082	34,002	32,740	32,032	8343 (40.9)	11637 (57.1)
- Internal Medicine	102,922	11,511	16,172	18,123	19,410	19,061	18,645	4661 (40.5)	7134 (62.0)
- Surgery	32,243	3,790	5,555	6,321	6,283	6,246	6,048	1765 (46.6)	2258 (59.6)
- Orthopaedics	20,471	2,597	3,354	3647	4075	3400	3,398	757 (29.1)	801 (30.8)
- Neurology	22,353	2,497	3,657	3991	4234	4033	3,941	1160 (46.5)	1444 (57.8)
Males	91,840 (51.0)	10,544 (51.7)	14,635 (50.9)	16,209 (50.5)	17,205 (50.6)	16,710 (51.0)	16,537 (51.6)		
Length of stay, mean (SD)	3:59 (6:32)	3:21 (4:15)	4:03 (5:25)	4:17 (12:01)	4:16 (4:35)	4:01 (4:11)	3:45 (4:22)		
Length of stay, median (IQR)	3:29 (2:06, 4:48)	2:37 (1:18, 3:37)	3:18 (1:58, 4:52)	3:26 (2:20, 5:03)	3:24 (2:29, 5:12)	3:14 (2:18, 4:59)	3:08 (2:02, 4:28)		
Length of stay ≤4 h	112,879 (62.7)	15,163 (74.3)	18,374 (63.9)	18,779 (58.5)	19,249 (56.6)	20,028 (61.2)	21,256 (66.4)		
Patients experiencing crowding	113,393 (63.0)	7,730 (37.9)	15,202 (52.9)	24,350 (75.9)	26,386 (77.6)	22,034 (67.3)	19,796 (61.8)

**Fig. 3:**
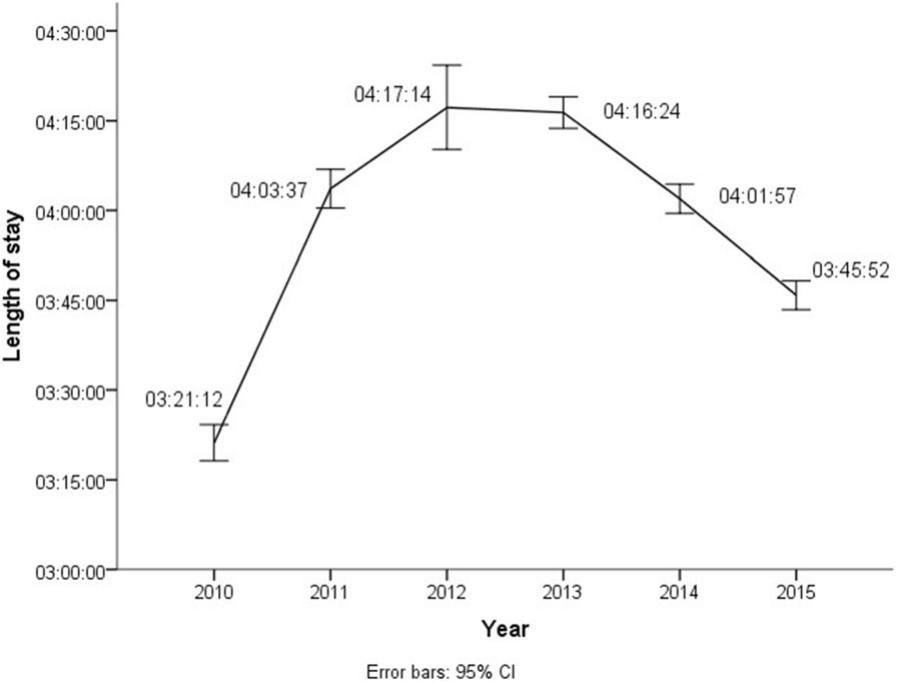
Emergency department patients ≥ 18 years, mean length of stay with 95% confidence interval error bars, for the period 2010–2015, *n* = 179,989

Mean LOS during the study period was 3:59 h (SD 6:32) (Table 2). In 2010, the average LOS was 3:21 h (SD 4:15). From 2010 to 2011, the average LOS increased by 42 min (20.9%), to 4:03 h (SD 5:25) (*p* < 0.001) (Table 2 and Fig. 3). For the 2011 ED population overall, the total LOS increased by 20 177 h from 2010 to 2011.

In the multiple linear regression model, all variables were significantly associated with LOS (Table 3). Regarding the association between LOS and admission year, there was a non-linear association, and 2010 was entered as the reference admission year in the model. All admission years 2011–2015 were significantly associated to LOS (*p* < 0.001), with an increasing regression coefficient from 2011 to 2013, and then declining to 2015.

**Table 3:** Multiple linear regression model with length of stay as the dependent variable

Variable	Regression coefficients (95% CI)	*P*-value
Admission year		
2010 – reference	0	
2011	42 (37; 47)	< 0.001
2012	53 (48; 59)	< 0.001
2013	55 (50; 61)	< 0.001
2014	38 (33; 43)	< 0.001
2015	19 (14; 25)	< 0.001
Admission day		
Monday – reference	0	
Tuesday	−5 (−10; 0)	0.038
Wednesday	−8 (−13; −3)	0.002
Thursday	−12 (−17; −7)	< 0.001
Friday	12 (7; 17)	< 0.001
Saturday	−40 (−45; −35)	< 0.001
Sunday	−35 (−41; −30)	< 0.001
Age group		
< 20 – reference	0	
20–29	38 (27; 49)	< 0.001
30–39	46 (35; 57)	< 0.001
40–49	45 (34; 56)	< 0.001
50–59	43 (32; 53)	< 0.001
60–69	49 (39; 60)	< 0.001
70–79	54 (44; 65)	< 0.001
80–89	62 (52; 73)	< 0.001
90+	63 (51; 74)	< 0.001
Gender		
Male – reference	0	
Female	11 (8; 13)	< 0.001
Specialty		
Internal medicine – reference	0	
Surgery	21 (17; 24)	< 0.001
Orthopaedics	13 (8; 17)	< 0.001
Neurology	−23 (−28; −19)	< 0.001

Regression coefficients and 95% CIs in minutes

Average LOS increased from 3.6 h (SD 4.7) during normal operational level, to 5.1 h (SD 6.6) during high operational level, and to 5.8 h (SD 6.3) during critical operational level.

**Fig. 4:**
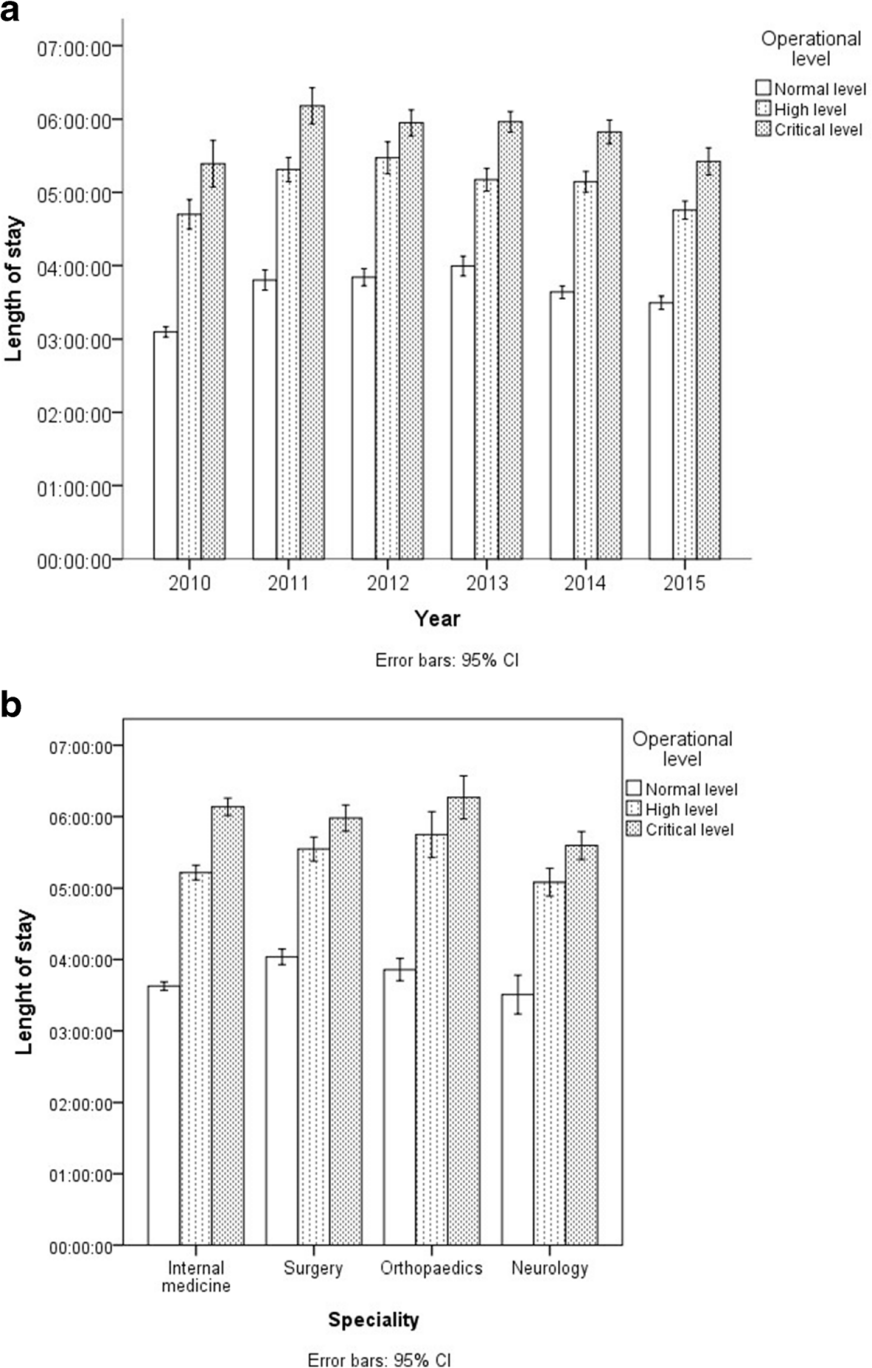
**a** Emergency department patients ≥ 18 years, mean length of stay with 95% confidence interval error bars for normal, high and critical operational levels for the period 2010–2015, *n* = 179,989. Normal operational level: < 25 / < 35 patients in the ED (2010/ 2011–2015). High operational level: 25–34/ 35–44 patients in the ED (2010/ 2011–2015). Critical operational level: ≥ 35/ ≥ 45 patients in the ED (2010/ 2011–2015). **b** Emergency department patients ≥ 18 years, mean length of stay with 95% confidence interval error bars for normal, high and critical operational levels per specialty (internal medicine, surgery, orthopaedics and neurology), *n* = 179,989. Normal operational level: < 25 / < 35 patients in the ED (2010/ 2011–2015). High operational level: 25–34/ 35–44 patients in the ED (2010/ 2011–2015). Critical operational level: ≥ 35/ ≥ 45 patients in the ED (2010/ 2011–2015)

Figure 4a shows the LOS for the three operational levels in the study period. The increase in LOS was present for all the four specialties. Figure 4b shows the LOS for normal, high and critical operational levels for each separate specialty.

**Fig. 5:**
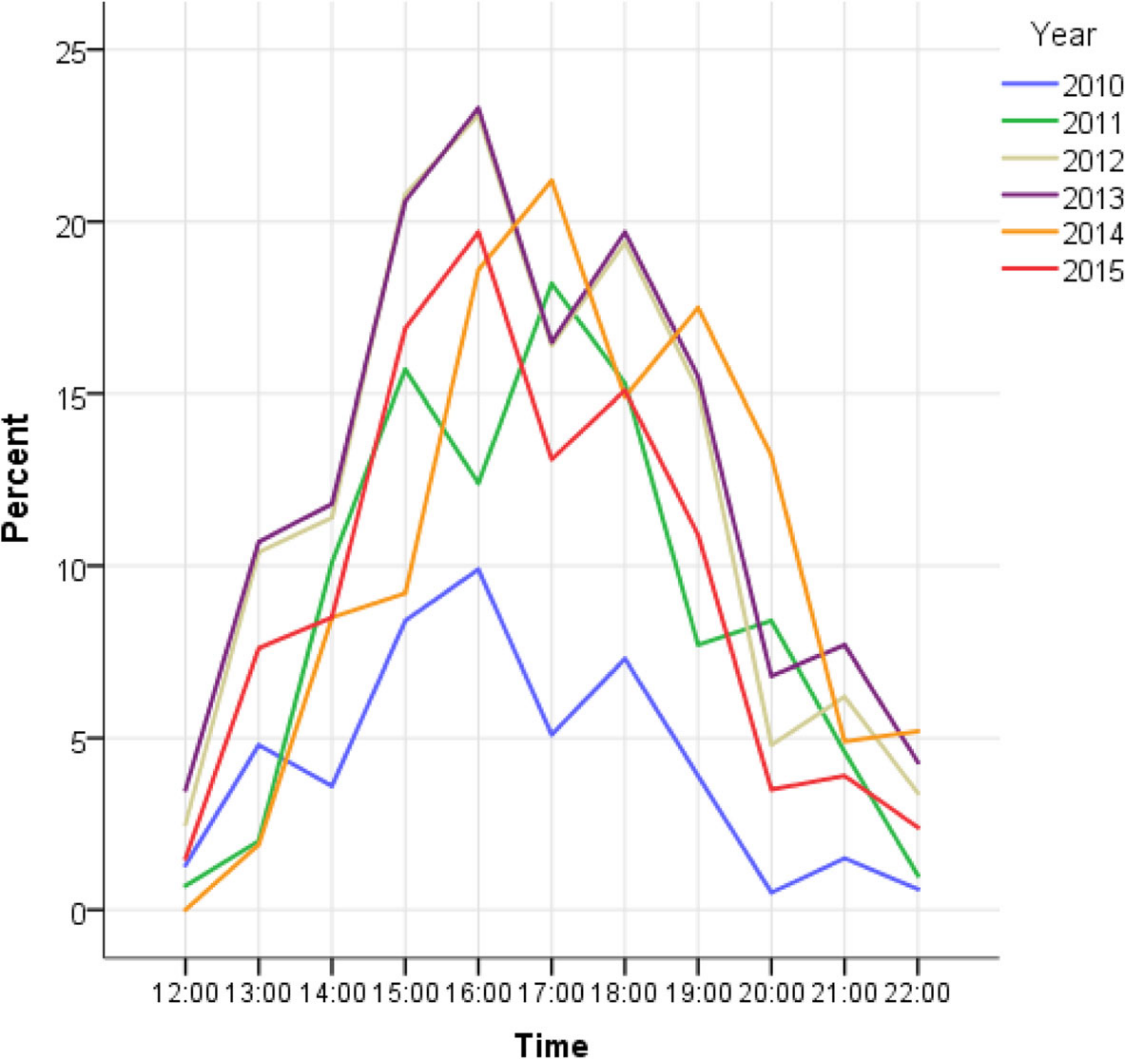
Percentage of emergency department patients experiencing crowding* between 12 AM and 10 PM per year, 2010–2015, *n* = 113,833. * High or critical operational level. High operational level: 25–34/ 35–44 patients in the ED (2010/ 2011–2015). Critical operational level: ≥ 35/ ≥ 45 patients in the ED (2010/ 2011–2015)

Overall, 37.9% of the patients admitted in 2010 experienced crowding (high or critical operational level) during their stay in the ED, and this proportion increased to 52.9% in 2011. Crowding peaked in 2013 (77.6%) (Table 2). Figure 5 and Additional file 1: Table S1 shows the percentage of patients per hour who experienced crowding between 12:00 and 22:00. Crowding peaked between 16:00–17:00 (Fig. 5).

## Discussion

The present study shows that LOS increased by 20.9% in Norway’s largest ED after hospital restructuring resulting in an increased catchment area, and overall, the total LOS increased by 20 177 h from 2010 to 2011. Even after 5 years, the LOS was higher than before the expansion, mainly because of the throughput and output components, which were not properly adapted to the changes in input. In the multiple linear regression analysis, 2010 was chosen as the reference admission year. All admission years 2011–2015 were significantly associated to LOS, but the model was not ideal because of a non-linear association between LOS and admission year.

Our findings are supported by previous studies [3, 4, 9]. An increase in ED LOS in times of crowding is not surprising considering that the number of patients who need acute care rises. A large increase in the number of patients combined with an increase in ED LOS leads to a mismatch in supply and demand. The need for emergency services exceeds the available resources [2]. Mondays were the day of the week with the highest volume of visits, like other studies done on crowding [13, 14]. Importantly, even 5 years after the increase in catchment area, in 2015, the LOS was higher compared to 2010.

In 2011, there was a hospital merger in the greater Oslo area, leading to Akershus University Hospital becoming the largest emergency care hospital in Norway. The present study shows that the increase in catchment area from about 340 000 to about 490 000 inhabitants increased the number of admissions to the ED by 40.9%. Norwegian EDs are organized by specialty, and the increase in patient volume varied between 29.1 and 46.5% between the different specialties. Because of the varying degree of increase, each specialty must consider their level of staffing and clinical experience in the ED. Overall, with a 44% increase in the catchment area, it is not surprising that the number of admissions increased by about the same figures. The mismatch in supply and demand is rather likely related to a lack of sufficient structural changes in the ED services, e.g. nursing cutbacks on top of a general nursing and physician shortage, and shortage of examination beds and rooms, in combination with other organizational barriers and inefficiencies within the hospital itself, such as shortage of inpatient bed capacity. Interestingly, despite gradual changes primarily in the throughput components, but also in the output components, the ED was not able to operate back to the levels of LOS and crowding even after 5 years compared to before the catchment area increase.

Crowding may also influence the prioritization of the patients. A study conducted in the Netherlands showed that crowding affected the triage process [15]. Times of crowding lead to a longer wait to be triaged and a prolonged ED LOS. The triage nurses did not meet their quality indicator of triaging patients within 10 min after registration, when crowding occurred. A growing waiting room was related to delays in triage and a lower degree of satisfaction [15]. Periods of crowding were related to under-classification and missing triage scores, possibly affecting patient outcome [15]. At Akershus University Hospital, triage was not introduced systematically until 2013, and is therefore not evaluated as an independent variable in this study. However, data show a breaking point about 2013 (LOS and crowding reduction from 2014), which partly could be related to the introduction of the MTS.

The Norwegian ED is difficult to compare to EDs in other countries because of different pre-hospital organization. In Norway, most patients are evaluated by their GP or by an EP before hospital admission, thus patients with low degree of urgency or severity, are not evaluated in the hospital. Advanced testing and more patients that are complex may affect ED LOS [4, 16]. The use of computer tomography (CT) scanning prolonged the length of stay among ED patients [2, 4, 17]. The average LOS seems to have a breaking point from 2013 to 2014 (cf. the multiple linear regression analysis), which probably, in addition to the introduction of the MTS, is related to an organizational change with an increase in the number of Internal medicine physicians in the ED from this year. Nursing staff increased by 7.9% in 2015. Studies show that adequate staffing is a plausible solution to ED crowding resulting in decreased LOS [18]. The shortage of critical care beds has also become a problem affecting the EDs. Critically ill patients ready to board have to wait in the ED for an inpatient bed. The acute care of these patients require a much higher degree of specialized treatment, and a large amount of resources are thus bound [4, 19]. EDs are not equipped or staffed to provide this kind of specialized care over a longer period of time. The delay in transfer might lead to increased morbidity and mortality among critically ill patients [4], which was not evaluated in this study. Patients waiting for an inpatient bed in regular or specialized wards are also an important factor, affecting the ED length of stay [5, 20, 21]. This applies especially to patients that require solitary confinements. Akershus University Hospital has had a high bed occupancy level, often about 100%. Hoot et al. [2] describe the connection between a hospital occupancy level above 90% and a substantially increase in ED LOS. Hospital occupancy is considered as an output component [9] in terms of crowding.

When the number of patients exeeded 45 patients in this study’s ED, in average every patient had an increased LOS of 2.2 h. Knowledge of the possible negative implications of ED crowding means that actions and initiatives must be taken in order to reduce this increased LOS. Both the United Kingdom and Australia have set up the “four-hour rule” in order to try to cope with the increased demands in ED care. The purpose is to make sure that 98% of all patients leave the ED within 4 h [22, 23]. A study conducted in Western Australia after the introduction of the “four-hour rule”, showed an improvement in regards of fewer events of ED crowding [22]. Moskop et al. [24] also supported the use of time limits to alleviate ED crowding. In our study, 74% of patients were discharged from the ED within 4 h in 2010, but this proportion decreased over the next few years. Importantly, most patients left the ED within 4 h in times of normal activity, but the problem of a prolonged LOS became an issue of concern already when the amount of patients reached the threshold level of 35. In a systematic review [2], the need of increased resources was underlined, which is in line with the results in this study, with average LOS decreasing in the last 2 years of the study period when physician and nurse staff were increased. Despite a development towards fewer days of crowding and a shorter LOS, Akershus University Hospital did not reach the LOS level prior to the increase in the catchment area. Moskop et al. [24] described a “reversed triage” as a solution to evacuate patients in times of crowding. “Reversed triage” made sure that the least critically ill patients were identified and evacuated, leaving the ED in capacity to treat critically ill patients. The use of advanced nursing interventions (blood samples and radiology referrals) in triage decreased the ED LOS, because patients spent less time waiting for test results [25].

The cause of an increased ED LOS is multifactorial. The results of this study did not differentiate between having to wait for triage, to be evaluated and treated by a physician or waiting for an inpatient bed (the different phases in the throughput component). Future studies should further investigate the factors contributing to ED crowding.

The findings in this study must be interpreted in the context of its limitations. This study was conducted in one single hospital and the results may not be generalizable to all ED settings. Data were collected retrospectively from the hospital’s electronic information system. The use of hospital records has limitations because the information is difficult to oversee retrospectively. The size of the dataset can be considered as a strength, because minor inaccuracies will not affect the overall results or time trends. The results of this study were not adjusted for hospital occupancy, which, according to other studies, might have an impact on ED LOS [9, 20, 24]. Future studies investigating ED LOS and crowding after hospital reorganization and mergers are needed to validate and generalize the findings of this study.

## Conclusion

The present study showed that the average LOS was prolonged in times of both high and critical operational levels in Norway’s largest emergency department, and that crowding was more frequent after a major increase in the hospital’s catchment area. The increased catchment area was a consequence of a bigger regional hospital merging, which aimed to reduce costs and increase quality. Increased LOS and crowding is often a sign of the opposite, as a longer stay in the ED increases the risk of adverse events and decrease patient safety.

The ED cannot solve the problem of crowding, as external factors such as changes to the catchment area or the health of the population admitted to the hospital. The response to ED crowding must therefore come from a higher institutional level. Organizational changes, which will cause an additional workload, such as an increased catchment area, must be thoroughly planned, and adequate resources must be invested in the throughput components from the beginning in order to not lie behind and the need to always catch-up with the difficulties.

## Additional file

Additional file 1: **Table S1.** Percentage of patients experiencing crowding between 12:00 and 22:00. (DOCX 13 kb)

## Data Availability

The data collected and analysed are not publicly available because of confidentially concerns. The Data Protection Authorities have not given permission to share data.

## Abbreviations

ED: Emergency department
EMS: Emergency medical services
EP: Emergency physicians
GP: General practitioner
LOS: Length of stay
MTS: Manchester Triage System
