# Supplementary material for: Emergency department crowding and length of stay before and after an increased catchment area
Source: BMC Health Serv Res. 2019 Jul 22;19:506. doi: 10.1186/s12913-019-4342-4 (PMC6647148; doi:10.1186/s12913-019-4342-4)
Supplement: Supplementary file 1 — Table S1. Percentage of patients experiencing crowding between 12:00 and 22:00. (DOCX 13 kb) [file 12913_2019_4342_MOESM1_ESM.docx]

Additional file 1: Table S1. Percentage of patients experiencing crowding between 12:00 and 22:00.

| **Time/Year** | **2010** | **2011** | **2012** | **2013** | **2014** | **2015** |
| --- | --- | --- | --- | --- | --- | --- |
| **12:00** | 1.3 | 0.7 | 2.5 | 3.5 | 0.0 | 1.5 |
| **13:00** | 4.8 | 2.0 | 10.4 | 10.7 | 1.9 | 7.6 |
| **14:00** | 3.6 | 10.1 | 11.4 | 11.8 | 8.5 | 8.5 |
| **15:00** | 8.4 | 15.7 | 20.8 | 20.6 | 9.2 | 16.9 |
| **16:00** | 9.9 | 12.4 | 23.1 | 23.3 | 18.6 | 19.7 |
| **17:00** | 5.1 | 18.2 | 16.4 | 16.5 | 21.2 | 13.1 |
| **18:00** | 7.3 | 15.3 | 19.4 | 19.7 | 14.9 | 15.1 |
| **19:00** | 3.9 | 7.7 | 15.1 | 15.5 | 17.5 | 10.9 |
| **20:00** | 0.5 | 8.4 | 4.8 | 6.8 | 13.2 | 3.5 |
| **21:00** | 1.5 | 4.6 | 6.2 | 7.7 | 4.9 | 3.9 |
| **22:00** | 0.6 | 1.0 | 3.4 | 4.3 | 5.2 | 2.4 |
